# Updated Clinical Practice Guidelines in Resuscitation and the Management of Respiratory Distress Syndrome in Extremely Preterm Infants during Two Epochs in Romania: Impact on Outcomes

**DOI:** 10.3390/jcm13041103

**Published:** 2024-02-15

**Authors:** Manuela Cucerea, Marta Simon, Mădălina Anciuc-Crauciuc, Raluca Marian, Monika Rusneac, Maria Livia Ognean

**Affiliations:** 1 Department of Neonatology, George Emil Palade University of Medicine, Pharmacy, Science, and Technology, 540142 Targu Mures, Romania; manuela.cucerea@umfst.ro (M.C.); madalina.anciuc@umfst.ro (M.A.-C.); 2 Department M1, Cellular and Molecular Biology, 540142 Targu Mures, Romania; raluca.marian@umfst.ro; 3 Clinical Department, Targu Mures Clinical and Emergency County Hospital, 540142 Targu Mures, Romania; monika.rusneac@yahoo.com; 4 Clinical Department, Faculty of Medicine, Lucian Blaga University Sibiu, 550169 Sibiu, Romania; maria.ognean@ulbsibiu.ro; 5 Neonatology Department, Sibiu Clinical and Emergency County Hospital, Lucian Blaga University Sibiu, 550245 Sibiu, Romania

**Keywords:** extremely preterm infants, resuscitation, neonatal respiratory distress syndrome, outcomes, guidelines

## Abstract

Background: Adequate perinatal management is essential in caring for extremely preterm (EP) infants. We aimed to evaluate and compare the impact of different protocols on short-term outcomes. Methods: A retrospective study was conducted on EP infants in a Romanian perinatal tertiary center during 2008–2012 and 2018–2022. Results: Data on 270 EP infants (121 in period I, 149 in period II) were analyzed collectively and stratified into two subgroups by gestational age. Initial FiO_2_ administration (100% vs. 40%% *p* < 0.001), lung recruitment at birth (19.0% vs. 55.7% *p* < 0.001), early rescue surfactant administration (34.7% vs. 65.8%; *p* < 0.001), and the mechanical ventilation rate (98.3% vs. 58.4%; *p* < 0.001) were significantly improved during period II. Survival rates of EP infants significantly improved from 41.3% to 72.5%, particularly in the 26–28 weeks subgroup (63.8% to 83%). Compared to period I, the overall frequency of severe IVH decreased in period II from 30.6% to 14.1%; also, BPD rates were lower (36.6% vs. 23.4%; *p* = 0.045) in the 26–28 weeks subgroup. Despite improvements, there were no significant differences in the frequencies of NEC, sepsis, PVL, ROP, or PDA. Conclusions: Implementing evidence-based clinical guidelines can improve short-term outcomes.

## 1. Introduction

Over recent decades, the survival of extremely preterm (EP) infants has increased since active strategies during transition and in the management of respiratory distress syndrome (RDS) were implemented and updated. Nevertheless, the increase in survival may lead to higher risks of neonatal morbidity among survivors, like intraventricular hemorrhage (IVH), periventricular leukomalacia (PVL), patent ductus arteriosus (PDA), necrotizing enterocolitis (NEC), early/late-onset sepsis (EOS, LOS), retinopathy of prematurity (ROP), bronchopulmonary dysplasia (BPD), cerebral palsy (CP), and neurodevelopmental impairment [1,2,3,4,5]. There is wide variability in morbidity rates for EP preterm infants due to variations in resuscitation initiation, administration of antenatal corticosteroids, delivery in tertiary centers, respiratory management, medical equipment, team training in the delivery room, and neonatal intensive care unit (NICU) and socioeconomic factors [6]. Thus, strategies to limit major complications of EP infants require special attention. Preterm infants are still at risk of experiencing hypoxia at birth and respiratory difficulties, the concerns of the clinicians being primarily focused on these aspects.

The European Resuscitation Council (ERC), the American Heart Association (AHA), and the American Academy of Pediatrics (AAP) have developed well-known resuscitation guidelines that are followed worldwide. The International Liaison Committee on Resuscitation (ILCOR) was founded in 1992 and publishes evidence-based resuscitation guidelines that reflect international consensus every five years (2000, 2005, 2010, 2015, 2020). Over the years, there have been many debates and controversies surrounding the stabilization of newborns, leading to significant changes in practice [7]. In the guidelines published in 2000 and 2005, elective intubation of EP infants was recommended in the delivery room (DR). The standard approach was to use 100% oxygen to initiate resuscitation in newborns with apnea or bradycardia, regardless of gestational age (GA). However, some preliminary evidence proposing resuscitation with lower oxygen concentrations has been published [7,8,9,10,11,12,13]. In 2015, the ILCOR guidelines specified that preterm infant resuscitation should be initiated with low-concentration oxygen or room air, monitored by pulse oximetry [14,15,16,17].

As for RDS, concerns about improving its management have led to the development of consensus recommendations based on a 3-year review of the most recent literature. The European Society for Paediatric Research (ESPR) endorsed the “European Consensus Guidelines for the Management of RDS” in 2007, which have evolved over the years (2010, 2013, 2016, 2019, and 2022). The 2007 edition recommended early intubation and prophylactic administration of natural surfactant to all newborn infants under 27 weeks of gestation, followed by mechanical ventilation (MV) or extubating to nasal continuous positive airway pressure (nCPAP) [18,19]. In 2013, the updated guidelines introduced significant changes in practice, including delaying clamping of the umbilical cord (DCC) for at least 60 s, initiating stabilization with 21–30% oxygen, stabilizing spontaneously breathing infants with RDS using nCPAP of at least 6 cm H_2_O, administering rescue surfactant early via the INSURE technique, avoiding MV if possible, and using early caffeine therapy. The guidelines also mention using a less invasive surfactant administration (LISA) method introduced in the 2019 and 2022 editions.

Neonatologists tried to enhance support for the adaptation to the extrauterine life of newborn infants by applying these recommendations. Before 2013, in our NICU, EP infants were prophylactically intubated and resuscitated with 100% oxygen, prophylactically treated with surfactant at birth, and received MV. We had no oxygen blender, but we used pulse oximetry for monitoring SpO_2_ without target saturation. After 2013, we introduced a locally updated protocol for resuscitation at birth and RDS management according to ILCOR, AHA, ESPR, and AAP recommendations. The purpose of the present study was to evaluate and compare the effects of different protocols on the early outcomes of EP infants in two periods separated by ten years of experience, education, training sessions, and endowment with high-performance equipment.

## 2. Materials and Methods

### 2.1. Study Group: Inclusion and Exclusion Criteria

**Figure 1:**
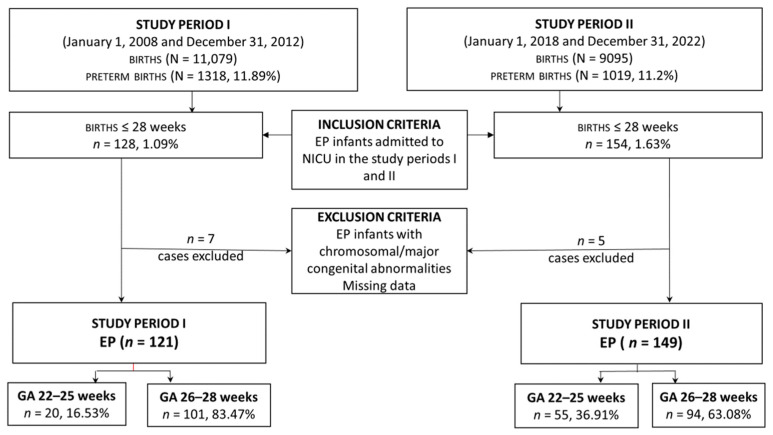
Study enrolment flow chart.

This retrospective study was conducted at Targu Mureș County Emergency Hospital, an academic perinatal level III center in Romania, over two different periods. The first period was from 1 January 2008 to 31 December 2012, and the second from 1 January 2018 to 31 December 2022. This study was approved by the hospital’s ethical committee (No. 6799/15.03.2022). Of 282 infants with a GA of 28 weeks or less admitted to NICU during both periods, 270 were considered eligible for the study. Infants with significant congenital and chromosomal anomalies, as well as those with missing data, were excluded. The infants were stratified into two subgroups for each study period based on their GA, 22–25 weeks, and 26–28 weeks (Figure 1).

### 2.2. Study Design and Data Acquisition

We studied and compared the two cohorts of EP infants managed with different protocols (Table 1) for delivery room stabilization and early management of RDS, analyzing the early outcomes (death before discharge and morbidity).

Data were collected retrospectively from the neonatal written medical NICU records. The following data were recorded for this study: maternal conditions (diabetes, hypertension, chorioamnionitis), prenatal care, antenatal steroid use (at least 2 doses of dexamethasone), premature rupture of membranes (PROM > 18 h), tocolysis, delivery location (inborn or outborn), delivery mode, gender, gestational age (evaluated according to the New Ballard Score), birth weight (BW), and small for gestational age (SGA) status (defined as BW below the 10th centile on Fenton’s growth chart).

The delivery room management data included Apgar scores, preductal peripheral oxygen saturation (SpO_2_) and oxygen concentration administered (FiO_2_) at 5 min, cord pH, nCPAP or positive pressure ventilation (PPV) use, intubation in the DR, DCC, and initial hematocrit (Hct).

The management for respiratory distress syndrome (RDS) data included the need for surfactant administration, surfactant prophylaxis (in the first 15 min after birth) or early rescue therapy (in the first two hours of life), mode of surfactant administration (conventional—via endotracheal tube, INSURE—extubation on nCPAP after surfactant replacement, LISA—via a thin catheter/feeding tube in spontaneously breathing infant on nCPAP), surfactant dose, need for MV at 72 h of life, duration of MV, and caffeine therapy. Curosurf (Poractant alpha, Chiesi Pharmaceuticals, Parma, Italy) was used in all patients eligible for surfactant administration.

Early outcome parameters collected included pneumothorax (PTX), BPD, IVH (all grades, grades 1–2 and 3–4), PVL, sepsis or probable sepsis, PDA, NEC, ROP stage ≥ 2, number of days spent in the NICU, and deaths. Bronchopulmonary dysplasia was defined as oxygen requirement at 36 weeks of post-menstrual age [20,21]. Intraventricular Hemorrhage was graded according to the criteria of Papile et al. [22]. Necrotizing enterocolitis was classified by Bell’s criteria [23]. Persistent Ductus Arteriosus diagnosis was based on clinical and echocardiographic parameters [24]. Retinopathy of prematurity was defined using the current ICROP classification [25]. Neonatal sepsis was identified by clinical, microbiological, hematological, and biochemical criteria for sepsis. Probable sepsis was defined as clinical signs, altered/abnormal inflammatory and hematological markers, and negative blood culture [26]. Mortality was defined as death before discharge from the maternity hospital. Criteria used for mechanical ventilation included: FiO_2_ > 0.4 to maintain SpO_2_ > 90%, or persistent apnea and/or respiratory distress. Severe neonatal morbidity was defined as a composite score of death, or either of the following: BPD, severe IVH, NEC, PVL, and/or severe ROP.

This study’s primary objective was to evaluate the impact of the changes in neonatal care at birth and RDS protocol in EP infants reflected by an improvement in the survival rate. The secondary objectives were (1) to evaluate the impact of the changes in neonatal care at birth and RDS protocol on the rate of morbidities associated with prematurity and (2) to define the factors and interventions with significant impact on mortality.

### 2.3. Statistical Analysis

The continuous variables were verified for normal distribution, and all were found to be abnormally distributed. Therefore, the Mann–Whitney U Test was used to compare the study groups. The Chi-square test and Pearson correlation were used to compare the categorical variables. The Kaplan–Meier survival analysis was used to calculate survival probabilities, and survival curves were generated for both groups. The log-rank test was then conducted to determine if there was a significant difference between the survival times of the groups, assuming the same conditions. Multivariable Cox regression was performed to identify the best predictors for mortality during the study period. The variables with significant impact on mortality were used to design the regression models, which were then used to predict mortality using receiver operating characteristics (ROC). The precision of the regression models was estimated by calculating the area under the ROC curve (AUC). The statistical significance level was set at *p*-value < 0.05. Odds ratios (ORs) were calculated where appropriate. IBM SPSS Statistics 26.0 was used for all statistical analyses.

## 3. Results

### 3.1. Baseline Demographic Characteristics

Data were collected from 121 EP infants in the 2008–2012 period (*n* = 20 for 22–25 weeks; *n* = 101 for 26–28 weeks) and from 149 infants in the 2018–2022 period (*n* = 55 for 22–25 weeks; *n* = 94 for 26–28 weeks). The baseline demographic characteristics of the two study cohorts are shown in Table 2.

In period I of the study, EP infants had a mean GA of 26.7 ± 1.1 weeks (24–28 weeks) compared to the mean GA of infants from period II, which was 26.1 ± 1.6 weeks (22–28 weeks). Significant differences were found between the two groups in terms of male gender (42.1% in period I vs. 57.7% in period II; *p* = 0.011), outborn infants (23.1% in period I vs. 12.8% in period II; *p* = 0.025), prenatal care (14% in period I vs. 61.1% in period II; *p* < 0.001), maternal hypertension (26.4% in period I vs. 8.7% in period II; *p* < 0.001), and delivery mode (vaginal—66.9% in period I vs. 50.3% in period II; *p* = 0.006) (Table 2).

The incidence of maternal complications such as diabetes and chorioamnionitis was similar in both study periods. The rates of antenatal steroid administration (54.5% vs. 61.1%) and tocolysis (14% vs. 17.4%) were not significantly different between groups (Table 2).

### 3.2. Delivery Room Stabilization

There was no significant difference in the Apgar score at 5 min between the two periods, as indicated in Table 3. However, the median umbilical cord pH was higher in period II (7.27 compared to 7.21 in period I; *p* = 0.002). Among the EP infants, only 23 (15.4%) received DCC in period II, and none received it in period I.

During period II, there was a significant reduction in the initial FiO_2_ used to stabilize EP infants. The FiO_2_ in period II ranged from 30 to 100%, with an average of 40.0%, compared to period I where it ranged from 50 to 100.0%, with an average of 100.0% (*p* < 0.001). Moreover, the SpO_2_ level at 5 min was lower in period II (80.0%) than in period I (83.0%; *p* < 0.001). In period II, nCPAP use for stabilization increased to 55.7%, while the use of PPV decreased to 49.7% compared to period I (19.0% and 74.4%, respectively). The percentage of infants intubated in the DR decreased significantly from 65.3% to 43.0% in the second period (*p* < 0.001). Significant improvements were found in the 26–28 weeks subgroup analysis during period II compared to period I. The improvements consisted of higher Agar score at 5 min (7 vs. 7.5; *p* = 0.020), increased umbilical cord pH (7.19 vs. 7.29; <0.001), and greater utilization of nCPAP (19.8% vs. 67.0%; *p* < 0.001). Additionally, lower administration of PPV (71.3 vs. 30.9%; *p* < 0.001) and intubation rates (58.4% vs. 23.4%; *p* < 0.001) were noted in this subgroup during period II. Significant differences were observed in the 22–25 weeks GA subgroup between study periods for SpO_2_ at 5 min (80% vs. 75%; *p* = 0.017) and intubation rate (100.0% vs. 76.4%; *p* = 0.016).

### 3.3. RDS Management (Surfactant and Mechanical Ventilation)

#### 3.3.1. Surfactant

In period II, a higher percentage of EP infants received surfactant (55.4% vs. 89.3%; *p* < 0.001) and early rescue surfactant treatment (34.7% vs. 65.8%; *p* < 0.001) compared to period I. No significant difference was found between the two groups in surfactant prophylaxis and other modes of surfactant administration (conventional and INSURE). The surfactant dose was higher in period II and LISA was performed in 39.6% of infants in the same period. Caffeine treatment was administered only in period II.

In the analysis of infants born at 26–28 weeks, during period II, a significant increase in overall surfactant administration (57.4% vs. 84%; *p* < 0.001), early rescue treatment (38.6% vs. 66.0%; *p* < 0.001), LISA method (0.0% vs. 52.1%; <0.001), and caffeine treatment (0.0% vs. 100.0%; <0.001) was noted as compared to period I. Conventional surfactant administration decreased in period II (42.6% vs. 12.8%; *p* < 0.001).

For infants born at 22–25 weeks, 98.2% received surfactant in period II, compared to 45% in period I. Early rescue treatment (15% vs. 65.5%; *p* < 0.001) and conventional surfactant administration (30.0% vs. 65.5%; *p* = 0.006) were also seen in more infants in period II. Table 4 shows early respiratory management in the study periods.

#### 3.3.2. Mechanical Ventilation

Infants born in period II required MV less often than those delivered in period I (98.3% vs. 58.4%; *p* < 0.001) at 72 h. EP infants born in both periods had similar lengths of MV (measured in hours) and were supported with similar ventilation modes (SIMV/HFOV). Volume-targeted ventilation (VTV) was only used in period II of the study, in 76.1% of ventilated infants.

When analyzing the subgroup of infants born between 26 and 28 weeks, significant improvements were observed during period II compared to period I. Need for MV at 72 h decreased (98.0% vs. 38.3%; *p* < 0.001), SIMV (59.6% vs. 32.2%; *p* = 0.042), and HFOV (40.4% vs. 21.6%; *p* = 0.042) were used in fewer cases. Infants born at 22–25 weeks had significantly longer MV duration in period II (312 days) compared to period I (48 days) (*p* = 0.001).

### 3.4. Outcomes (Survival/Deaths and Morbidities)

The comparison of morbidities and deaths between the study periods is presented in Table 5.

The group of EP infants born between 2018 and 2022 had significantly lower rates of intraventricular hemorrhage (all IVH) (26.2%) and severe IVH (14.1%) when compared to the group of EP infants born between 2008 and 2012. This difference was statistically significant (*p* = 0.002 and *p* = 0.001, respectively). However, there was no significant difference between the two groups in the rates of other health conditions such as PTX, BPD, NEC, sepsis/probable sepsis, and severe ROP.

During period I, 41.3% of infants admitted to our NICU died. However, the death rate decreased significantly to 27.5% (*p* = 0.017) during period II. Additionally, the early death rate was significantly lower during period II (15.4% vs. 25.6%; *p* = 0.038) than in period I.

We noted significant improvements in the outcomes in the 26–28 gestational weeks subgroup when comparing the two study periods. Thus, the rates of BPD (36.6% vs. 23.4%; *p* = 0.045), all IVH (26.7 vs. 17.0%; *p* < 0.001)/severe IVH (26.7% vs. 4.3%; *p* < 0.001), deaths (36.2% vs. 17.0%; *p* = 0.002), and early deaths (21.8% vs. 9.6%; *p* = 0.020) were lower in period II compared to period I.

Although not statistically significant, improvements were also found in the 22–25 gestational weeks subgroup when comparing the two study periods: lower rates of all IVH (65.0% vs. 41.8%), severe IVH (50.0% vs. 30.9%), PVL (15.0% vs. 7.3%), PDA (40.0% vs. 29.1%), deaths (65.0% vs. 45.5%), early deaths (40.0% vs. 25.5%).

**Figure 2:**
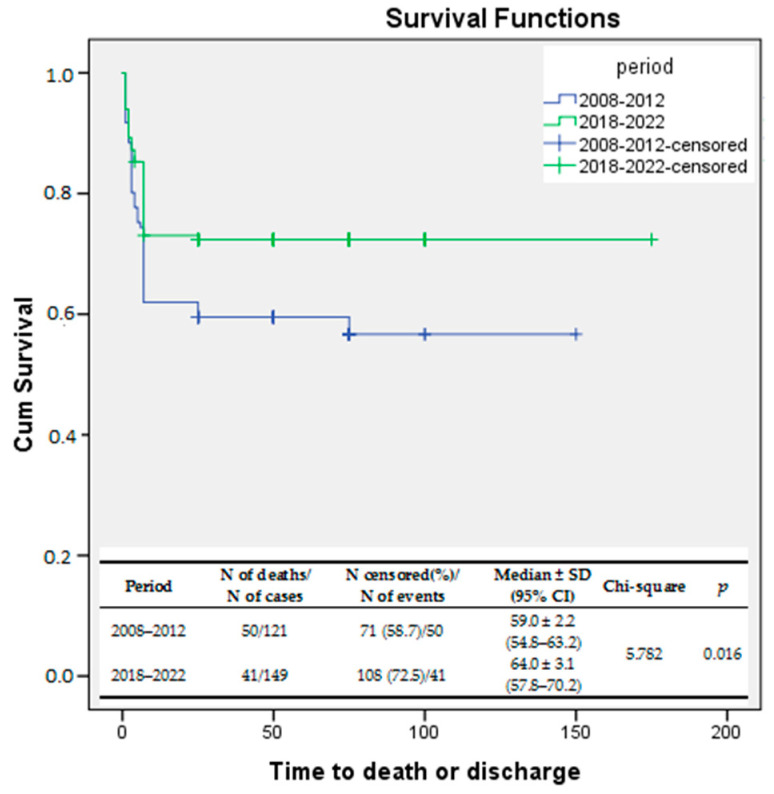

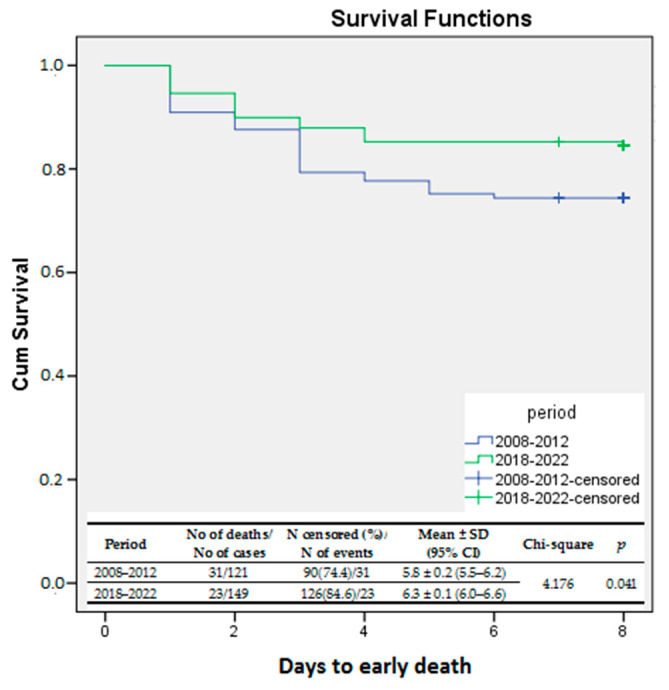
Kaplan–Meier survival curves—survival rates compared between the study periods.

Kaplan–Meier survival curves were used to evaluate the survival rates during the two study periods. The log-rank test was used to compare the survival rates of the two study periods. The results showed a significant improvement in the survival rate during the second period of the study compared to the first one (log-rank *p* = 0.016). The same statistical analysis was performed for early death (0–6 days of life). Early survival rates were significantly improved in EP infants born between 2018 and 2022 compared to those delivered between 2008 and 2012 (log-rank *p* = 0.041). (Figure 2).

**Figure 3:**
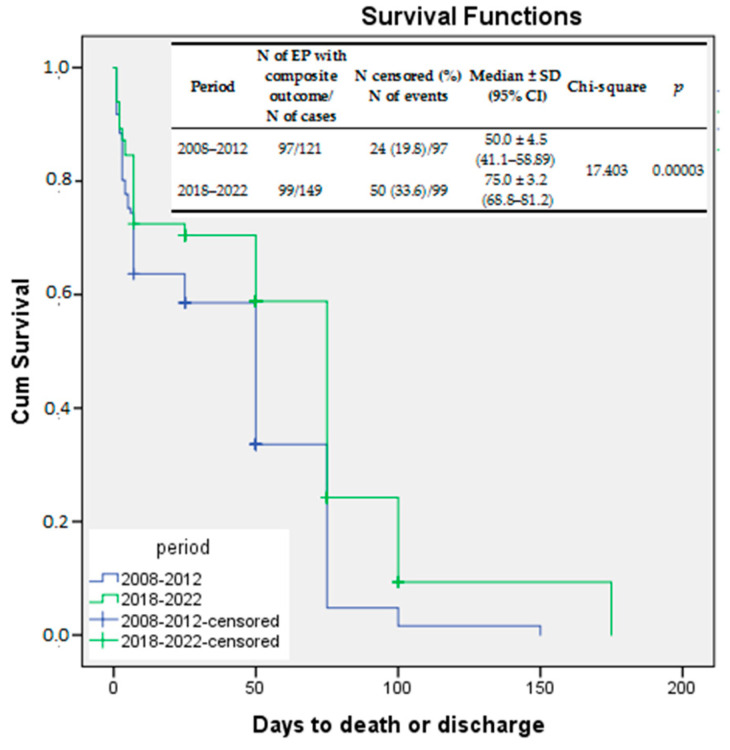
Kaplan–Meier survival curves—survival free of significant morbidities compared between the study periods.

Kaplan–Meier survival curves were also used to compare the survival rates without any significant risk for long-term neurodevelopmental impairment. The variable tested was the composite outcome (death or major IVH ± PVL ± NEC ± severe ROP), and the log-rank test compared the survival rates without long-term risk for neurodevelopmental outcome between the two study periods. Again, a significant improvement was demonstrated for EP infants born between 2018 and 2022 as compared to those born between 2008 and 2012 (log rank *p* = 0.00003) (Figure 3).

**Figure 4:**
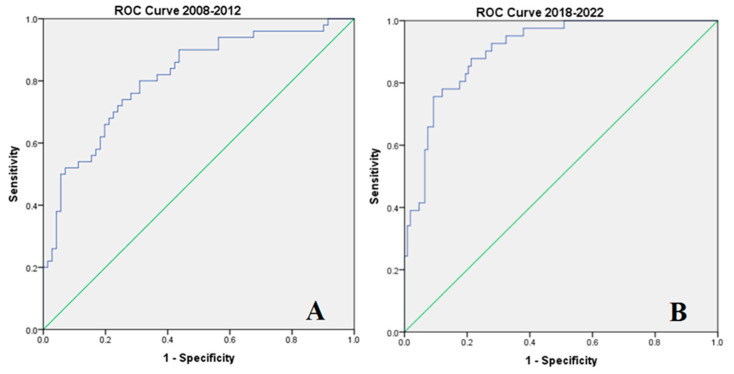
ROC curves were built using the mortality prediction models as evaluated by multivariable Cox regression: (**A**) for 2008–2012, (**B**) for 2012–2022. ROC curves (AUC delineated by blue lines) were built using the mortality prediction models as evaluated by multivariable Cox regression: (**A**) for 2008–2012, (**B**) for 2012–2022 (in green x = y).

Multivariate Cox regression was used to build prediction models for mortality in the two birth study periods (Table 6). Each model—including statistically significant variables in Cox regression—was tested on the receiver operating curve to test the predictive power of the model (Figure 4A,B). As seen in Table 6 and in Figure 4, the predictive models using the variables with statistical significance had strong predictive power even though different variables influenced mortality during the study periods (AUC for 2008–2012 = 0.811, AUC for 2018–2022 = 0.907, *p* < 0.001 for both).

This study has identified several factors that increase the risk of mortality in infants. During the first period, these factors included low birth weight, lack of antenatal steroids, postnatal transfer of EP infants, severe intraventricular hemorrhage (IVH), and bronchopulmonary dysplasia (BPD). In the second period, BPD and NEC had a negative impact on the survival rate, while alveolar recruitment and surfactant administration decreased the risk of death.

## 4. Discussion

Ensuring survival and the best possible outcomes for EP infants are crucial goals for healthcare professionals. This study is the first in Romania to analyze resuscitation trends in the delivery room, the early management of RDS in EP infants based on guidelines, and their impact on neonatal outcomes. Our research has shown that overall survival rates of EP infants have increased from 41.3% (2008–2012) to 72.5% (2018–2022), despite a lower mean GA in the second study period. The most significant improvement was observed in the 26–28 weeks subgroup, where the survival rate increased from 63.8% to 83%. The survival rate for the subgroup of 22–25 weeks of gestation improved from 35% to 54.5% in period II. In Romania, the viability limit is 24 weeks of gestation, but our unit has been proactively resuscitating from 22 weeks since 2016. During period II, the overall early death rate was lower, with the most significant decrease seen in the 26–28-weeks subgroup, where only 9.4% of infants died, compared to 21.8% in period I. Previously published literature on survival and short-term outcomes among EP infants has shown variable results due to variable proactive resuscitation of periviable infants. Survival rates in our study are slightly lower than those reported by Bell et al. [5], who conducted a study of a large cohort of infants with the same GA (78.3%). According to a study conducted by Higgins in California (2011–2019) on infants ≤ 28 weeks, the survival rate of infants increases with each week of gestation, ranging from 33% at 22 weeks to 90% at 28 weeks [27].

Improved perinatal care and resuscitation algorithms, according to the new 2015 and 2020 International Consensus on neonatal resuscitation [15,16] in period II of the study, have led to better outcomes for EP infants. The improvements include in-utero transfer, delayed cord clamping, targeted SpO_2_ to avoid high FiO_2_ administration, avoiding intubation and PPV in the delivery room, and using non-invasive nCPAP for lung recruitment. In the last reporting period, 87.2% of EP infants treated in our NICU were inborn, 61.1% received prenatal care, and 49.7% were delivered via C-section. Delayed umbilical cord clamping was documented only for 15.4% of EP infants, less than reported in other studies [5], although this procedure brings many benefits [28]. It is recommended to perform DCC in preterm infants who do not require extensive resuscitation. Delayed cord clamping rates vary between 6.7 and 46.7% for EP infants in different European regions [29]. However, infants born before 28 weeks of gestation may require medical interventions at birth, and it may not always be feasible to collaborate with obstetricians or possess the specific equipment to provide resuscitation while the umbilical cord is still attached.

At 5 min, the Apgar score and cord pH were higher, while FiO_2_ (median 40%) and SpO_2_ at 5 min (80%) were lower during the second study period. Additionally, during period II, changes in resuscitation practice led to a 40% decrease in the use of endotracheal intubation (43.0%) and a 47% decrease in PPV (49.7%). Instead, nCPAP was used 2.75 times more often than in period I. We found that alveolar recruitment was significantly associated with survival, as was in-utero transfer. Our results were consistent with those of a recent study reporting that over half of newborns required intubation at birth, while the percentage of positive pressure ventilation without intubation was 22.9% [30].

During period II, the management of RDS was improved in accordance with updated guidelines. One of the key changes was the administration of high-dose early rescue surfactant through less invasive methods. Additionally, where possible, a non-invasive mode of ventilation was used, and lung-protective ventilation was provided when needed. Mechanical ventilation was avoided as much as possible, and caffeine treatment was also utilized. The length of stay in the NICU was 22 days, longer in period II, which can be attributed to the lower gestational age of the infants.

During the second period studied, there was a significant rise in the use of surfactant replacement for RDS. Among EP infants, 89.3% received surfactant and 65.8% early rescue administration. LISA was the preferred mode of surfactant replacement in 39.6% of infants, particularly in the 26–28 weeks subgroup. Consequently, the requirement for MV at 72 h of life decreased from 98.3% to 58.4% in all EP infants. In the 26–28 weeks subgroup, the requirement decreased even further, to 38.3%. The mean duration of invasive ventilation was 259.4 ± 234.2 h in period II, higher than in period I. Volume-targeted ventilation was utilized in 76.1% of the ventilated EP infants, while caffeine was administered to all infants.

Although there have been improvements in stabilizing infants in the delivery room and early management of RDS, BPD still affected a significant number of infants at 30.9% between 2018 and 2022. It is likely that the higher survival rates of infants born before 25 weeks of GA contributed to this. Low gestational age is a significant risk factor for bronchopulmonary dysplasia (BPD). However, reported rates of BPD vary widely between studies depending on the inclusion criteria and local practice guidelines. Norman and colleagues reported that the incidence of bronchopulmonary dysplasia (BPD) was between 79 and 49% in infants born at 23 and 26 weeks of gestational age (GA), respectively [31]. In a recent study, severe BPD was observed in 9.6–37.0% of preterm infants born before 28 weeks of GA and who survived until discharge [6]. Obstetric interventions, such as high rates of in-utero transfer, antenatal steroids, tocolytic treatment, intrapartum antibiotherapy, cerclage, and cesarean section, should be considered to reduce the risk of BPD in these high-risk infants [32]. However, we have observed a significant decrease in the frequency of BPD among infants born between 26 and 28 weeks of GA.

In our study, the frequency of all IVH and severe IVH decreased in period II among survivors of EP infants from 43.8% to 26.2% and from 30.6% to 14.1%, respectively. We did not observe any significant improvements in PVL (8.1%), NEC at all stages (22.8%), PDA (31.5%), sepsis/probable sepsis (21.5%), and ROP ≥ 2 (18.1%) rates between study periods. A Turkish study reported rates of severe IVH of 14.5%, severe NEC of 4.6%, PDA of 50.7%, late-onset sepsis of 24.0%, and severe ROP of 13.3% among ELBW infants between 2017 and 2021 [33].

The prevalence of the composite outcome (major neonatal morbidity ± death) in our study remained high, at 66.4%, although it decreased significantly compared to the first period.

Kaplan–Meier survival curves clearly confirmed the improvements in survival, early survival (0–6 days of life), and survival without significant morbidities associated with continuous updates of the protocols for resuscitation at birth and care of EP with RDS. Multivariable Cox regression has shown differences in factors influencing the mortality rate in the two periods of study as different factors influenced the risk of death of EP infants born between 2008 and 2012 compared to those delivered between 2018 and 2022.

Our study has provided insight into the impact of improved strategies on early outcomes of EP infants, by offering an image of practice according to guidelines during two periods. Due to the study’s retrospective design, limited sample size, missing stillbirth data, and lack of comprehensive follow-up assessment, certain limitations may be considered when interpreting the results. Nevertheless, the findings of this study provide valuable insights into the topic, at least for Romanian neonatology, but further multicentric research is needed to have a better overview of the outcomes of EP infants and to identify strategies for further improvements.

This study also highlights the importance of enhancing perinatal and postnatal care for extremely premature infants and identifying other risk factors that may lead to unfavorable outcomes. We can conduct further research and develop more effective interventions and proactive measures to improve outcomes by identifying these factors.

The significant improvement in the survival and mortality rate clearly indicates the efficacy of our actions and the progress we have made in ensuring a better outcome. It is important to consider the benefits of evidence-based strategies and protocols for the stabilization and management of RDS. By implementing these strategies, we can provide the best possible care for these fragile and at-risk populations of newborns, increasing their chances of survival with fewer complications. The future concerns of clinicians should be focused on the development of new strategies to approach preterm infants born at the limit of viability.

## 5. Conclusions

New guidelines for resuscitation and early management of respiratory distress syndrome have improved survival rates and short-term outcomes for EP infants born between 2018 and 2022 compared to 2008–2012. Training healthcare professionals and updated guidelines and protocols are crucial for better outcomes.

## Figures and Tables

**Table 1:** Essential differences between protocols in the studied periods.

	Period I (2008–2012)	Period II (2018–2022)
Delivery room respiratory management		
Neonatal resuscitation algorithm	2005/2010	2015/2020
Delayed cord clamping	No	In infants who do not require extensive resuscitation
FiO_2_ (%) for initiating resuscitation	100% (<100% since 2011)	30–100%
SpO_2_ (%) at 5 min	No target SpO_2_	Target SpO_2_ 80–85%
PPV	Elective	Non-breathing preterm infants
nCPAP (lung recruitment)	No/yes, since 2012, PEEP 5 cmH_2_O	Yes, PEEP > 6 cm H_2_O
Intubation in the delivery room	Elective intubation	When needed
RDS management		
Antenatal steroids	Yes	Yes
Surfactant prophylaxis	Yes	Only in the special situation
Rescue Surfactant	Yes	Early rescue—the first option
Dose of surfactant	100–200 mg/kg	200 mg/kg
Surfactant delivery	Conventional/INSURE	LISA/INSURE
nCPAP	after MV	First intention
MV modes	Conventional, HFOV	Lung-protective ventilation VTV
Caffeine	No	Yes

DCC: Delayed cord clamping; FiO_2_ (%): Fraction of Inspired Oxygen; SpO_2_ (%): peripheral oxygen saturation; PPV: Positive Pressure Ventilation—bag and mask/T-piece resuscitator; HFOV: High-Frequency Oscillatory Ventilation; VTV: Volumetargeted ventilation; INSURE: INtubation-SURfactant administration-Extubation; LISA: Less Invasive Surfactant Administration.

**Table 2:** Baseline demographic characteristics.

	Study Group (*n* = 270)		*p*-Value	OR
	Period I 2008–2012 (*n* = 121) (*n* = 20; 22–25 w) (*n* = 101; 26–28 w)	Period II 2018–2022 (*n* = 149) (*n* = 55; 22–25 w) (*n* = 94; 26–28 w)		
Male (*n*/%) ^†^ 22–25 w 26–28 w	51/42.1 6/30.0 45/44.6	86/57.7 32/58.2 54/57.4	0.011 * 0.031 * 0.773	1.41 (1.08–1.85) 2.39 (1.03–2.56) 1.28 (0.98–1.89)
GA, weeks median (IQR) ^‡^ 22–25 w 26–28 w	27 (26–28) 25 (25–25) 27 (26–28)	26 (25–27.5) 24 (24–25) 27 (26–28)	<0.001 * <0.001 * 0.814	-
BW, g, median (IQR) ^‡^ 22–25 w 26–28 w	880 (750–960) 650 (512.5–797.5) 900 (800–980)	800 (650–990) 620 (590–700) 950 (800–1000)	0.114 0.488 0.190	-
SGA, (*n*/%) ^†^ 22–25 w 26–28 w	20/16.5 6/30.0 14/13.9	18/12.1 1/1.8 17/18.1	0.298 <0.001 * 0.423	0.83 (0.59–1.16) 0.24 (0.14–0.42) 1.17 (0.78–1.78)
Singleton (*n*/%) ^†^ 22–25 w 26–28 w	113/93.4 18/90.0 95/94.1	126/84.6 43/78.2 83/88.3	0.024 * 0.251 0.156	0.38 (0.17–0.90) 0.48 (0.13–185) 0.66 (0.34–1.28)
Outborn (*n*/%) ^†^ 22–25 w 26–28 w	28/23.1 1/5.0 27/26.7	19/12.8 3/5.5 16/17.0	0.025 * 0.939 0.103	0.69 (0.48–0.99) 1.02 (0.57–1.83) 0.72 (0.48–1.10)
Prenatal care (*n*/%) ^†^ 22–25 w 26–28 w	17/14.0 4/20.0 13/12.9	91/61.1 27/49.4 64/68.1	<0.001 * 0.024 * <0.001 *	4.08 (2.60–6.40) 2.82 (1.04–7.62) 4.42 (2.66–7.33)
Maternal hypertension (*n*/%) ^†^ 22–25 w 26–28 w	32/26.4 8/40.0 24/23.8	13/8.7 2/3.6 11/11.7	<0.001 * <0.001 * 0.028*	0.56 (0.43–0.71) 0.23 (0.13–0.42) 0.70 (0.53–0.92)
Maternal diabetes (*n*/%) ^†^ 22–25 w 26–28 w	6/5.0 1/5.0 5/5.0	4/2.7 0/0 4/4.3	0.327 0.098 0.818	0.72 (0.44–1.24) - 0.93 (0.51–1.69)
Chorioamnionitis (*n*/%) ^†^ 22–25 w 26–28 w	10/8.3 4/20.0 6/5.9	15/10.1 7/12.7 8/8.6	0.601 0.438 0.477	1.14 (0.69–1.87) 0.67 (0.28–1.67) 1.23 (0.66–2.29)
Prenatal steroids (*n*/%) ^†^ 22–25 w 26–28 w	66/54.5 11/55.0 55/54.5	91/61.1 32/58.2 59/62.8	0.281 0.809 0.242	1.16 (0.89–1.51) 1.10 (0.52–2.33) 1.18 (0.90–1.54)
Tocolysis (*n*/%) ^†^ 22–25 w 26–28 w	17/14.0 5/25.0 12/11.9	26/17.4 10/18.2 16/17.0	0.450 0.520 0.339	1.16 (0.78–1.72) 0.75 (0.32–1.73) 1.24 (0.79–1.95)
PROM > 18 h (*n*/%) ^†^ 22–25 w 26–28 w	35/28.9 5/25.0 30/29.7	45/30.2 19/34.5 26/27.7	0.820 0.440 0.754	1.03 (0.77–1.39) 1.41 (0.58–3.43) 0.95 (0.71–1.28)
Vaginal delivery (*n*/%) ^†^ 22–25 w 26–28 w	81/66.9 13/65.0 68/67.3	75/50.3 26/47.3 49/52.1	0.006 * 0.179 0.030 *	0.67 (0.50–0.90) 0.58 (0.26–1.30) 0.73 (0.54–0.98)

Continuous variables were expressed as follows: median (IQR); categorical variables: number (%); *n*: number; w: weeks; GA: gestational age; BW: birth weight; SGA: small for gestational age; PROM: preterm rupture of membranes; ^†^ Chi-square test; IQR: interquartile range; ^‡^ Mann–Whitney U Test; * Marked effects are significant at *p* < 0.05.

**Table 3:** Delivery room stabilization.

	Study Group (*n* = 270)		*p*-Value	OR
	Period I 2008–2012 (*n* = 121) (*n* = 20; 22–25 w) (*n* = 101; 26–28 w)	Period II 2018–2022 (*n* = 149) (*n* = 55; 22–25 w) (*n* = 94; 26–28 w)		
DCC (*n*/%) ^†^ 22–25 w 26–28 w	0/0 0/0 0/0	23/15.4 4/7.3 19/20.2	<0.001 * 0.331 <0.001 *	-
Apgar score 5 min, median (IQR) ^‡^ 22–25 w 26–28 w	7 (5–8) 6.5 (6.25–7) 7 (6–8)	7 (5–8) 5 (4–7) 7.5 (6–8)	0.883 0.513 0.020 *	-
Cord pH median (IQR) ^‡^ 22–25 w 26–28 w	7.21 (6.98–7.30) 7.23 (6.98–7.28) 7.19 (6.98–7.31)	7.27 (7.12–7.32) 7.21 (7.10–7.28) 7.29 (7.21–7.33)	0.002 * 0.871 <0.001 *	-
FiO_2_ (%), median (IQR) ^‡^ 22–25 w 26–28 w	100 (50–100) 100 (40–100) 100 (50–100)	40 (30–100) 77.5 (40–100) 40 (30–60)	<0.001 * 0.409 <0.001 *	-
SpO_2_ (%) 5 min, median (IQR) ^‡^ 22–25 w 26–28 w	83 (77.5–88) 80 (69–87.5) 83 (78–88)	80 (70–86) 75 (67–80) 82 (77.5–88)	0.004 * 0.017 * 0.541	-
nCPAP ≥ 6 cmH_2_O (*n*/%) ^†^ 22–25 w 26–28 w	23/19.0 3/15.0 20/19.8	83/55.7 20/36.4 63/67.0	<0.001 * 0.078 <0.001 *	2.75 (1.87–4.03) 2.51 (0.81–7.72) 3.00 (2.01–4.47)
PPV (*n*/%) ^†^ 22–25 w 26–28 w	90/74.4 18/90.0 72/71.3	74/49.7 45/81.8 29/30.9	<0.001 * 0.400 <0.001 *	0.53 (0.38–0.74) 0.58 (0.16–2.19) 0.43 (0.31–0.60)
Intubation (delivery room) (*n*/%) ^†^ 22–25 w 26–28 w	79/65.3 20/100.0 59/58.4	64/43.0 42/76.4 22/23.4	<0.001 * 0.016 * <0.001 *	0.60 (0.45–0.80) - 0.51 (0.38–0.67)
HGB (g/dL) at birth median (IQR) ^‡^ 22–25 w 26–28 w	15.2 (14.2–16.45) 14.8 (13.65–17.07) 15.3 (14.3–16.45)	15.5 (14.7–16.65) 15.8 (14.7–16.8) 15.4 (14.67–16.5)	0.058 0.097 0.346	-

Continuous variables were expressed as follows: median (IQR); categorical variables: number (%); *n*: number; w: weeks; PPV: positive pressure ventilation; DCC: delayed cord clamping; HGB: Hemoglobin; ^†^ Chi-square test; IQR: interquartile range; ^‡^ Mann–Whitney U Test; * Marked effects are significant at *p* < 0.05.

**Table 4:** RDS management.

	Study Group (*n* = 270)		*p*-Value	OR
	Period I 2008–2012 (*n* = 121) (*n* = 20; 22–25 Weeks) (*n* = 101; 26–28 Weeks)	Period II 2018–2022 (*n* = 149) (*n* = 55; 22–25 Weeks) (*n* = 94; 26–28 Weeks)		
Surfactant				
Surfactant dose (mg/kg), median (IQR) ^‡^ 22–25 w 26–28 w	100 (100–120) 120 (100–120) 100 (100–120)	200 (200–200) 200 (200–200) 200 (200–200)	<0.001 * <0.001 * <0.001 *	-
Surfactant administration (*n*/%) ^†^ 22–25 w 26–28 w	67/55.4 9/45.0 58/57.4	133/89.3 54/98.2 79/84.0	<0.001 * <0.001 * <0.001 *	2.30 (1.82–2.91) 6.42 (3.42–12.03) 1.75 (1.37–2.24)
Surfactant prophylaxis (*n*/%) ^†^ 22–25 w 26–28 w	25/20.7 6/30.0 19/18.8	35/23.5 18/32.7 17/18.1	0.580 0.826 0.897	1.10 (0.79–1.53) 1.10 (0.48–2.50) 0.98 (0.69–1.38)
Rescue Surfactant (*n*/%) ^†^ 22–25 w 26–28 w	42/34.7 3/15.0 39/38.6	98/65.8 36/65.5 62/66.0	<0.001 * <0.001 * <0.001 *	2.03 (1.52–2.70) 6.14 (1.96–19.21) 1.71 (1.28–2.27)
Conventional surfactant (*n*/%) ^†^ 22–25 w 26–28 w	49/40.5 6/30.0 43/42.6	48/32.2 36/65.5 12/12.8	0.160 0.006 * <0.001 *	0.82 (0.63–1.07) 2.97 (1.28–6.88) 0.53 (0.42–0.67)
INSURE (*n*/%) ^†^ 22–25 w 26–28 w	18/14.9 3/15.0 15/14.9	26/17.4 8/14.5 18/19.1	0.571 0.961 0.426	1.11 (0.76–1.63) 0.97 (0.34–2.78) 1.17 (0.78–1.74)
LISA (*n*/%) ^†^ 22–25 w 26–28 w	0/0 0/0 0/0	59/39.6 10/18.2 49/52.1	<0.001 * 0.041 * <0.001 *	- - -
Caffeine (*n*/%) ^†^ 22–25 w 26–28 w	0/0 0/0 0/0	149/100.0 55/100.0 94/100.0	<0.001 <0.001 <0.001	- - -
Mechanical ventilation strategies				
Need of MV at 72 h (*n*/%) ^†^ 22–25 w 26–28 w	119/98.3 20/100.0 99/98.0	87/58.4 51/92.7 36/38.3	<0.001 0.221 <0.001	0.05 (0.01–0.21) - 0.04 (0.01–0.18)
SIMV (*n*/%) ^†^ 22–25 w 26–28 w	69/58.0 10/50.0 59/59.6	49/55.7 20/39.2 28/32.2	0.742 0.415 0.042	-
HFOV (*n*/%) ^†^ 22–25 w 26–28 w	50/42.0 10/50.0 40/40.4	39/44.3 31/60.8 8/21.6	0.742 0.415 0.042	1.04 (0.82–1.32) 1.37 (0.65–2.86) 0.80 (0.66–0.98)
VTV (*n*/%) ^†^ 22–25 w 26–28 w	0/0 0/0 0/0	67/76.1 44/86.3 23/62.2	<0.001 * <0.001 * <0.001 *	-
Duration of MV, hours, median (IQR) ^‡^ 22–25 w 26–28 w	148 (49–345) 48 (17–172) 196 (73–372)	220 (72–408) 312 (90–480) 124.5 (35.2–238.5)	0.299 0.001 * 0.072	-

w: weeks; SIMV: Synchronized Intermittent Mandatory Ventilation; HFOV: High-Frequency Oscillatory Ventilation; VTV: Volume-targeted ventilation; INSURE: INtubation-SURfactant-Extubation; LISA: Less Invasive Surfactant Administration; Continuous variables were expressed as follows: median (IQR); categorical variables: number (%); *n*: number; IQR: interquartile range; ^†^ Chi-square test; IQR: interquartile range; ^‡^ Mann–Whitney U Test; * Marked effects are significant at *p* < 0.05.

**Table 5:** Short-term outcomes and mortality—comparison between the study groups.

	Study Group (*n* = 270)		*p*-Value	OR
	2008–2012 (*n* = 121) (*n* = 20; 22–25 w) (*n* = 101; 26–28 w)	2018–2022 (*n* = 149) (*n* = 55; 22–25 w) (*n* = 94; 26–28 w)		
PTX, *n* (%) 22–25 w 26–28 w	13/10.7 2/10.0 11/10.9	8/5.4 4/7.3 4/4.3	0.102 0.705 0.083	0.70 (0.49–1.01) 0.78 (0.27–2.60) 0.68 (0.49–0.96)
BPD, *n* (%) 22–25 w 26–28 w	41/33.9 4/20.0 37/36.6	46/30.9 24/43.6 22/23.4	0.600 0.063 0.045 *	0.93 (0.70–1.22) 2.38 (0.88–6.42) 0.75 (0.57–0.98)
NEC, *n* (%) 22–25 w 26–28 w	21/17.4 4/20.0 17/16.8	34/22.8 15/27.3 19/20.2	0.269 0.528 0.546	1.22 (0.84–1.76) 1.36 (0.52–3.56) 1.12 (0.77–1.63)
IVH all, *n* (%) 22–25 w 26–28 w	53/43.8 13/65.0 40/39.6	39/26.2 23/41.8 16/17.0	0.002 * 0.077 <0.001 *	0.66 (0.51–0.86) 0.50 (0.22–1.11) 0.61 (0.48–0.79)
IVH grade 3–4 (severe), *n* (%) 22–25 w 26–28 w	37/30.6 10/50.0 27/26.7	21/14.1 17/30.9 4/4.3	0.001 * 0.131 <0.001 *	0.62 (0.48–0.80) 0.56 (0.27–1.18) 0.52 (0.42–0.64)
PVL, *n* (%) 22–25 w 26–28 w	17/14.0 3/15.0 14/13.9	12/8.1 4/7.3 8/8.5	0.114 0.316 0.240	0.74 (0.52–1.03) 0.58 (0.23–1.51) 0.79 (0.56–1.12)
PDA, *n* (%) 22–25 w 26–28 w	52/43.0 8/40.0 44/43.6	47/31.5 16/29.1 31/33.0	0.053 0.377 0.130	0.77 (0.59–0.99) 0.71 (0.33–1.50) 0.81 (0.62–1.06)
Sepsis/probable sepsis, *n* (%) 22–25 w 26–28 w	33/27.3 7/35.0 26/25.7	32/21.5 13/23.6 19/20.2	0.270 0.332 0.362	0.85 (0.63–1.13) 0.67 (0.31–1.45) 0.86 (0.64–1.16)
ROP ≥ 2 *n* (%) 23–25 w 26–28 w	22/18.2 5/25.0 17/16.8	27/18.1 16/29.1 11/11.7	0.990 0.731 0.310	0.99 (0.71–1.41) 1.17 (0.48–2.81) 0.83 (0.59–1.16)
NICU, days, median (IQR) ^†^ 22–25 w 26–28 w	16 (5.5–26) 8.5 (2.25–30.25) 17 (7–26)	22 (14–32) 24 (10–35) 22 (15–31)	0.002 * 0.082 0.005 *	-
Deaths, *n* (%) 22–25 w 26–28 w	50/41.3 13/65.0 37/36.2	41/27.5 25/45.5 16/17.0	0.017 * 0.138 0.002 *	0.72 (0.56–0.94) 0.55 (0.25–1.23) 0.65 (0.50–0.83)
Early death (0–6 days) (*n*/%) 22–25 w 26–28 w	31/25.6 9/45.0 22/21.8	23/15.4 14/25.5 9/9.6	0.038 * 0.107 0.020 *	0.73 (0.55–0.96) 0.54 (0.26–1.12) 0.68 (0.51–0.89)
Composite outcome (N/%) 22–25 w 26–28 w	97/80.2 18/90.0 79/78.2	99/66.4 49/89.1 50/53.2	0.012 * 0.912 <0.001 *	0.65 (0.46–0.94) 0.93 (0.26–3.29) 0.68 (0.51–0.89)

Continuous variables were expressed as follows: median (IQR); categorical variables: number (%); *n*: number; w: weeks; PTX: pneumothorax; BPD: bronchopulmonary dysplasia; NEC: necrotizing enterocolitis; IVH: intraventricular hemorrhage; PVL: periventricular leukomalacia; PDA: patent ductus arteriosus; ROP: retinopathy of prematurity; NICU: Neonatal Intensive Care Unit; Composite outcome = death or IVH grade 3–4 ± LPV ± BPD ± NEC ± severe ROP (≥2); IQR: interquartile range; ^†^ Mann–Whitney U Test, * Marked effects are significant at *p* < 0.05.

**Table 6:** Results of the multivariable Cox regression.

	2008–2012 Period			2012–2022 Period		
	*p*	95.0% CI		*p*	95% CI	
		Lower	Upper		Lower	Upper
GA	0.643	0.746	1.608	0.744	0.691	1.303
BW	0.037 *	0.995	1.000	0.955	0.998	1.002
Prenatal steroids	0.005 *	1.315	4.790	0.131	0.846	3.616
Outborn	0.042 *	0.161	0.969	0.142	0.060	1.499
Vaginal delivery	0.593	0.600	2.444	0.318	0.716	2.798
Apgar at 5 min	0.206	0.943	1.313	0.494	0.736	1.159
Alveolar recruitment	0.749	0.439	3.142	0.006 *	1.458	9.665
Intubation at birth	0.491	0.562	3.317	0.172	0.688	8.139
Surfactant	0.129	0.001	2.476	0.031 *	0.001	0.730
Severe IVH	0.001 *	0.130	0.609	0.133	0.223	1.219
BPD	0.000 *	3.356	26.648	0.000 *	6.017	79.616
NEC	0.061	0.964	4.980	0.015 *	1.197	5.401
Surfactant dose	0.145	0.942	1.009	0.031 *	0.971	0.999
AUC	0.811 ± 0.040	0.733	0.889	0.907 ± 0.024	0.859	0.955
Chi-square	97.609			122.368		
*p*	<0.001			<0.001		

Legend: AUC—area under curve; * Marked effects are significant at *p* < 0.05.

## Data Availability

The data presented in this study are available on request from the corresponding authors.
